# TLR2-Bound Cancer-Secreted Hsp70 Induces MerTK-Mediated Immunosuppression and Tumorigenesis in Solid Tumors

**DOI:** 10.3390/cancers17030450

**Published:** 2025-01-28

**Authors:** Ahmet Kaynak, Subrahmanya D. Vallabhapurapu, Harold W. Davis, Eric P. Smith, Petr Muller, Borek Vojtesek, Robert S. Franco, Wen-Hai Shao, Xiaoyang Qi

**Affiliations:** 1 Division of Hematology & Oncology, Department of Internal Medicine, College of Medicine, University of Cincinnati, Cincinnati, OH 45267, USA; kaynakat@ucmail.uc.edu (A.K.); subrahmaya@gmail.com (S.D.V.); harold.davis19@gmail.com (H.W.D.); smithep4554@gmail.com (E.P.S.); francors@ucmail.uc.edu (R.S.F.); 2 Masaryk Memorial Cancer Institute, Research Centre for Applied Molecular Oncology, Zluty Kopec 7, 656 53 Brno, Czech Republic; pmuller@post.cz (P.M.); vojtesek@mou.cz (B.V.); 3 Division of Rheumatology, Allergy & Immunology, Department of Internal Medicine, College of Medicine, University of Cincinnati, Cincinnati, OH 45267, USA; shaowi@ucmail.uc.edu

**Keywords:** Hsp70, phospho-Hsp70, TLR2, MerTK, M2 macrophage polarization, cancer

## Abstract

**Background**: A hallmark of cancer is the presence of an immunosuppressive tumor microenvironment (TME). Immunosuppressive M2 macrophages (MΦs) in the TME facilitate escape from immune surveillance and promote tumor growth; therefore, TME-induced immunosuppression is a potent immunotherapeutic approach to treating cancer. **Methods**: Cancer cell-secreted proteins were detected by using liquid chromatography–mass spectrometry (LC-MS). Neutralizing antibodies (nAbs) were used to assess which proteins were involved in MΦs polarization and differentiation. The protein–protein interaction was characterized using co-immunoprecipitation and immunofluorescence assays. Cancer-secreted heat shock protein 70 (Hsp70) protein was quantified using an enzyme-linked immunosorbent assay (ELISA). MΦ polarization and tumor growth were assessed in vivo with subcutaneous LLC-GFP tumor models and toll-like receptor 2 (TLR2) knockout mice; in vitro assessments were conducted using TLR2 knockout and both LLC-GFP and LN227 lentiviral-mediated knockdown (KD) cells. **Results**: Cancer cells released a secreted form of Hsp70 that acted on MΦ TLR2 to upregulate Mer receptor tyrosine kinase (MerTK) and induce MΦ M2 polarization. Hsp70 nAbs led to a reduction in CD14 expression by 75% in THP-1 cells in response to Gli36 EMD-CM. In addition, neutralizing TLR2 nAbs resulted in a 30% and 50% reduction in CD14 expression on THP-1 cells in response to MiaPaCa-2 and Gli36 exosome/microparticle-depleted conditioned media (EMD-CMs), respectively. Hsp70, TLR2, and MerTK formed a protein complex. Tumor growth and intra-tumor M2 MΦs were significantly reduced upon cancer cell Hsp70 knockdown and in TLR2 knockout mice. **Conclusions**: Cancer-secreted Hsp70 interacts with TLR2, upregulates MerTK on MΦs, and induces immunosuppressive MΦ M2 polarization. This previously unreported action of secreted Hsp70 suggests that disrupting the Hsp70-TLR2-MerTK interaction could serve as a promising immunotherapeutic approach to mitigate TME immunosuppression in solid cancers.

## Simple Summary

Despite tremendous progress over the past 50 years, there remains an urgent medical need to develop novel approaches to treat malignancies. Identifying new targets essential for tumor growth is a key to further advances. An opportunity to identify additional mediators of cancer for which innovative therapies could be developed is to expand understanding of the immunosuppressive tumor microenvironment (TME). In this study, we discovered a previously unknown non-chaperone function of cancer-secreted heat shock protein 70 (Hsp70): the promotion of immunosuppressive macrophage (MΦ) M2 polarization. Specifically, Hsp70, secreted by cancer cells, interacted with toll-like receptor 2 (TLR2) and triggered Mer receptor tyrosine kinase (MerTK) upregulation to stimulate MΦ M2 polarization and tumor growth. Further studies will focus on how targeting Hsp70 may inhibit cancer growth by reactivating the immune system.

## 1. Introduction

Cancer cells have evolved mechanisms to establish an immunosuppressive tumor microenvironment (TME) that promotes escape from host immune attack and promotes tumor growth [1,2,3,4]. The reversal of this immunosuppression is a powerful approach to cancer treatment [1,3,5,6]. The TME is a complex milieu comprised of many secreted factors and diverse cell types, including tumor-associated macrophages (MΦs) and myeloid-derived suppressor cells (MDSCs), which are known to be the predominant cellular mediators of immunosuppression [1,5,7,8]. Circulating monocytes and resident MΦs are recruited to the tumor site, where tumor-derived factors promote their maturation into immunosuppressive M2 MΦs that enhance tumor progression [5,9,10,11,12,13,14].

In this study, we investigated the nature of factors secreted by cancer cells that induce MΦ M2 polarization. Proteins in cancer cell-conditioned media were surveyed, and the predominant MΦ polarizing activity could be accounted for by secreted heat shock protein 70 (Hsp70). Interestingly, Hsp70 is one of many proteins implicated in the promotion of cancer cell growth [15,16,17,18]. Although Hsp70 was initially discovered as an intracellular chaperone protein involved in the cellular stress response, it is overexpressed in a variety of cancers [17,18,19,20]. Increased plasma membrane-bound and circulating-Hsp70 have been detected in patients with glioblastoma, pancreatic, and lung cancer [16,20,21]. Furthermore, the depletion of Hsp70 has been shown to reduce tumor growth in pancreatic ductal adenocarcinoma, glioblastoma, colon, prostate, and hepatocellular carcinomas [17,19,20]. Hsp70 lacks an orthodox secretory signal, and its secretion is thought to occur non-conventionally, involving either lysosomal endosomes or by association with membrane rafts and other secretory proteins [22,23,24,25,26,27]. Post-translational modifications of Hsp70, such as phosphorylation, play critical roles in the chaperone function [28,29,30] and, more speculatively, may affect extracellular action on cancer [28].

Unlike intracellular Hsp70, the function of cancer-secreted Hsp70 on tumor growth is not well understood, partly because the extracellular receptor-mediated mechanisms of action are obscure. Although Hsp70 is known to bind toll-like receptors (TLRs), a family of receptors that are well-documented for their pro-inflammatory behavior [31,32,33,34,35], the functional implications are unclear. However, damage-associated molecular patterns (DAMPs) released by tumorigenic cells are also recognized by TLRs on MΦ, and this DAMP-TLR axis is known to regulate the immune response in TME [36]. TLRs, expressed abundantly on MΦ, bind specifically to a broad spectrum of bacterial or pathogen structures triggering inflammatory responses [33,34,35]. This suggests that Hsp70 may also affect inflammatory pathways involving MΦs. Moreover, the role of MΦ TLRs in regulating tumor-induced immunosuppression is also unclear. TAM (Tyro3, Axl, and Mer) receptor tyrosine kinases play a crucial role in MΦ M2 polarization in the TME [37,38], but it has not been determined whether TLRs communicate with these receptors in the TME to exert MΦ M2 polarization.

This report demonstrates that Hsp70, present in exosome/microparticle-depleted conditioned media (EMD-CM), interacted with TLR2 to induce a marked upregulation of MerTK. As MerTK is directly linked to MΦ M2 polarization and immunosuppression [37,38], our finding suggests a new mechanism for enhancing tumor immunosuppression.

## 2. Materials and Methods

### 2.1. Cell Lines

The cell lines studied were primary human astrocytes (ScienCell Research Laboratories, Carlsbad, CA, USA), primary human pancreatic ductal epithelial (HPDE) (CVCL_0P38) cells, human pancreatic cancer cell lines (AsPC-1 (ATCC-CRL-1682), MiaPaCa-2 (ATCC-CRL-1420), cfPac1-Luc3), melanoma cell line (A2058) (ATCC-CRL-3601), lung cancer cell line (H1299 (ATCC-CRL-5803), LLC-GFP), and glioblastoma cell lines (Gli36, U373 (ATCC HTB-17), U87ΔEGFR, LN229 (ATCC-CRL-2611)). Several human and mouse macrophage models have been used to study the effect of cancer cell EMD-CM on macrophage differentiation and polarization. These include human leukemia monocytic cell line THP-1 (ATCC-TIB202) and a normal human monocyte cell line SC (ATCC-CRL-3622). Using primary macrophage cell lines has several advantages including that they are homogenous and straightforward to propagate and maintain in the laboratory. TLR2 knock-out THP-1 cells generated using CRISPR/Cas9 were a kind gift from Dr. Veit Hornung to Dr. Qi and were described previously [39]; Gli36 and U87ΔEGFR cells were a gift from Dr. Balveen Kaur to Dr. Qi; the human pancreatic cfPac1-Luc3 cells were a kind gift of Dr. O. Wildner to Dr. Qi; LLC-GFP (CSC-RRO525, Creative Biogene Inc., Shirley, NY, USA) cells were a kind from Dr. John C. Morris (University of Cincinnati, Cincinnati, OH, USA) to Dr Qi.

### 2.2. Cell Culture

Wild-type (WT) and TLR2 null THP-1 cells, AsPC-1, MiaPaCa-2, cfPac1-Luc3, and human SC cells were cultured in RPMI with 25 mM HEPES. HPDE cells were cultured in a keratinocyte cell medium (Sigma-Aldrich, St. Louis, MO, USA) supplemented with the provided growth factor supplements, FBS and antibiotics. All other cell lines were cultured in DMEM, and all cell culture media were supplemented with 10% FBS and 1% penicillin/streptomycin. All cells were grown in a 5% CO_2_ incubator at 37 °C. Cells were routinely tested for mycoplasma contamination. No cross-contamination was observed in the cell lines, as determined using cellular morphology and growth parameters.

### 2.3. Generation of Mouse Bone Marrow-Derived MΦ (BMDM)

Mouse bone marrow was obtained from tibias and femurs of C57BL/6J mice (7–8 weeks old). After erythrocyte lysis, mouse bone marrow-derived MΦ (BMDM) was generated by growing cells in RPMI supplemented with mouse MCSF (PeproTech, Rocky Hill, NJ, USA) for 10 days with fresh cytokine-supplemented medium every three days.

### 2.4. Generation of Human Monocyte-Derived MΦ (HMDM)

Human peripheral blood monocytes were purchased from Zen-Bio Inc.,Durham, NC. Human monocyte-derived MΦ (HMDM) was generated by culturing monocytes in RPMI supplemented with human MCSF (PeproTech, NJ, USA) for 10 days. Fresh media and cytokines were added every three days. 

### 2.5. MΦ Polarization of BMDM and HMDM

BMDM was polarized into M2 MΦs by culturing with mouse IL-4 and IL-10 (10 ng/mL; PeproTech, NJ, USA) for 3 days. HMDM was polarized into the M2 phenotype through culture with human IL-4 and IL-13 (10 ng/mL; PeproTech, Rocky Hill, NJ, USA) for 3 days. M1 polarization was induced through the culture of BMDM and HMDM with mouse and human IFNγ, respectively (PeproTech, Rocky Hill, NJ, USA). Alternatively, BMDM and HMDM were cultured with the EMD-CMs obtained from human cancer cells. 

### 2.6. Generation of EMD-CM from Human/Mouse Cancer Cell Lines and THP-1 Differentiation Assay

Human/mouse cancer cell lines were grown in their respective media until ~70% confluency in 10 cm Corning tissue culture plates (ThermoFisher, Waltham, MA, USA), when the media were removed. The cells were washed twice with serum-free media to remove remnants of serum and dead cells, and replenished with serum-free media. After 24 h, conditioned media were collected and centrifuged at 10,000× *g* to remove cellular debris, followed by ultracentrifugation at 100,000× *g* to remove extracellular exosomes and microparticles to generate EMD-CM. THP-1 cells (2 × 10^5^) were cultured in EMD-CMs from indicated cancer cell lines for 24 h. Control THP-1 cells were grown in DMEM for 24 h. THP-1 cells, treated with 200 ng/mL phorbol 12-myristate-13-acetate (PMA; EMD Millipore Corporation, Burlington, MA, USA) for 48 h, were used as a positive control. Controls and EMD-CM-treated cells were centrifuged and then incubated with anti-CD14-PE-conjugated antibody (eBioscience, San Diego, CA, USA) and 7-Aminoactinomycin D (7AAD; Invitrogen, Waltham, MA, USA) in 100 µL FACS buffer for 30 min on ice. Cells were washed with flow cytometry buffer (PBS with 2% FBS), and CD14 expression was analyzed using flow cytometry.

### 2.7. LC-MS and THP-1 Culture in the Presence of Neutralizing Antibodies (nAbs)

For mass spectrometry analysis, serum-free media were added to Gli36 or MiaPaCa-2 cells for 24 h. 2 mL of CM was collected. Serum-free media were used as a control. Both CM and serum-free media were centrifuged at 100,000× *g* for 1 h min at 4 °C, and then passed through a 100 kDa filter. After filtration, the EMD-CM and serum-free media were incubated with trypsin at room temperature for 15 min. Finally, the EMD-CM and serum-free media were submitted for LC-MS analysis at the Mass Spectrometry Laboratory core facility at the University of Cincinnati. Samples were analyzed with a nano LC-MS/MS on a Sciex 5600 quadrupole-TOF system. The specific protein species were identified using the homo sapiens databases with the Protein Pilot (ver 5.0, rev 4769) program (Sciex, Framingham, MA, USA), as described previously [40]. 

For neutralization studies involving TLRs, THP-1 cells were cultured with EMD-CM obtained from human cancer cell lines for 24 h, in the presence or absence of nAbs (1 µg/mL; eBioscience, San Diego, CA, USA) against TLR2. For another control group, we used IgG at the same concentration as the IgG antibody. For neutralization studies involving Hsp70, enolase, moesin, SSA, and control IgG, THP-1 cells were incubated with 200 ng of antibody (Santa Cruz Biotechnology, Dallas, TX, USA) in 1 mL of culture medium. After 24 h, the THP-1 cells were harvested using centrifugation, stained with PI to exclude dead cells and with anti-CD14, and analyzed using flow cytometry.

### 2.8. Flow Cytometry Analysis

THP-1, SC, BMDM, or tumor-derived MΦs were washed once with PBS. Cells were then incubated with respective antibodies in flow cytometry buffer (PBS with 2% FBS) on ice. After 45 min of incubation, cells were washed twice with the flow cytometry buffer. THP-1 differentiation was assessed using flow cytometric measurement of PE-conjugated anti-CD14 (eBioscience, San Diego, CA, USA). For mouse M2 polarization, either M2-polarized BMDM or EMD-CM-treated BMDM were stained with anti-mouse F4/80-FITC for the total macrophage population (eBioscience, San Diego, CA, USA) and mouse M2 specific markers anti-CD206-APC and anti-CD301-FITC (BioLegend, San Diego, CA, USA). 7AAD was added to all stained cells to gate out dead cells. Stained cells were analyzed with a BD Fortessa X-20 flow cytometer (BD Biosciences, Cambridge, UK). 

### 2.9. Subcutaneous Cancer Cell Implantation

Six-week-old WT and TLR2-deficient C57BL/6J male mice (Jackson Laboratories, Bar Harbor, ME, USA) were subcutaneously injected with Lewis lung carcinoma cells (1 × 10^5^) expressing green fluorescent protein (LLC-GFP). For the Hsp70 knockdown (KD) experiment, WT and Hsp70 KD LLC-GFP cells (1 × 10^5^) were implanted subcutaneously into 6–8-week-old C57BL/6J male mice. Tumor growth was assessed daily by measuring the tumor volume. When the volume reached 500 mm^3^, the tumors were collected and used for histology and tumor MΦ analyses. Tumor volumes were measured using vernier calipers and calculated using the formula V = (π/6) LW2 (V, volume; L, length; W, width).

### 2.10. Tumor Dissociation into Single Cells and Flow Cytometry

Freshly excised tumors were cut into small pieces and minced using a scalpel. The minced tumor tissue was incubated with 100 units of collagenase type 4 (Worthington Biochemicals, Lakewood, NJ, USA) for 45 min at 37 °C in RPMI medium. The collagenase-treated tumor tissue was passed through a 40 µm cell strainer (ThermoFisher, Waltham, MA, USA). The isolated tumor cells were washed twice with PBS, and 5 × 10^5^ cells were incubated in 100 µL flow cytometry buffer (PBS with 2% FBS) with mouse Fc block for 30 min on ice, followed by incubation with anti-mouse F4/80-PE (eBioscience, San Diego, CA, USA) and anti-mouse CD206 APC (BioLegend, San Diego, CA, USA) for 30 min on ice. Cells were washed and incubated with fixation buffer (eBioscience, San Diego, CA, USA) for 30 min. Fixed cells were centrifuged and washed twice with PBS. Cells were permeabilized using permeabilization buffer (eBioscience, San Diego, CA, USA) according to the manufacturer’s protocol and then incubated with anti-mouse iNOS antibody (eBioscience, San Diego, CA, USA) in permeabilization buffer for 45 min. Cells were washed twice with a permeabilization buffer and finally washed with a flow cytometry buffer. MΦs were analyzed for CD206^+^ M2 cells and iNOS^+^ M1 phenotypes after gating on F4/80 positivity. 

### 2.11. Lentiviral-Mediated Knockdown (KD) of Hsp70

The pLKO.1 vectors expressing shRNAs targeting Hsp70 were obtained from Sigma-Aldrich (St. Louis, MO, USA). The pLKO.1 lentiviruses were packaged in HEK-293T cells by co-transfecting the pMD2.G (VSV G) envelope plasmid and the Gag, Pol-expressing psPAX2 packaging plasmid. These cells were cultured for 48 h after transfection, and the lentiviral particles were collected from the supernatants and used to transduce LLC-GFP cells. Gene silencing efficiency was analyzed by immunoblotting for Hsp70 at 36 h post-infection.

### 2.12. Immunoprecipitation Analyses

SC cells were incubated overnight with 10× concentrated MiaPaCa-2 EMD-CM (obtained by passing MiaPaCa-2 EMD-CM through a 10 kDa molecular weight cutoff filter) at 4 °C for 24 h. Immunoprecipitation was carried out using IgG (Santa Cruz Biotechnology, Dallas, TX, USA) or anti-MerTK monoclonal antibody (mAb) (Cell Signaling Technology, Danvers, MA, USA). The mixture was incubated at 4 °C overnight, followed by the addition of protein AG beads. The beads were centrifuged and washed twice with cell lysis buffer, re-suspended in SDS loading buffer, and analyzed using Western blot for Hsp70 using anti-Hsp70 mAb (Santa Cruz Biotechnology, Dallas, TX, USA) and TLR2 using anti-TLR2 mAb (Cell Signaling Technology, Danvers, MA, USA).

### 2.13. Immunofluorescence Staining

Cells were seeded on gelatin (0.01%)-coated coverslips. After 5 h incubation, cells were washed twice with PBS and then fixed with 4% formaldehyde for 10 min at room temperature. After fixation, cells were washed two times with PBS. For Hsp70 staining, cells were stained with the primary anti-Hsp70 antibody (Abcam, Cambridge, UK) followed by anti-rabbit IgG (H + L) and F(ab’)2 fragment AlexaFluor 555 conjugated (Cell Signaling Technologies, Danvers, MA, USA). For MerTK staining, cells were stained with primary anti-MerTK antibody (Santa Cruz Biotechnology, Santa Cruz, CA, USA) followed by anti-mouse IgG (H + L) and F(ab’)2 fragment AlexaFluor 647 conjugated (Abcam, Cambridge, UK). For TLR2 staining, cells were stained with primary anti-TLR2 antibody (Abcam, Cambridge, UK) followed by anti-rabbit IgG (H + L) and F(ab’)2 fragment AlexaFluor 488 conjugated (Abcam, Cambridge, UK). Cells were further stained with FITC-conjugated annexin V (ABP Biosciences, Rockville, MD, USA). After washing, the coverslips were mounted using the anti-fade Fluoro-gel II with DAPI (Electron Microscopy Sciences, Hatfield, PA). Negative controls were stained with only anti-rabbit IgG or mouse IgG-FITC (Santa Cruz Biotechnology, CA, USA). Samples were analyzed using a BX51 fluorescence microscope (Olympus, Tokyo, Japan) and a BioTek Lionheart FX digital microscope (BioTek, Winooski, VT, USA).

### 2.14. SDS-PAGE and Western Blotting Analyses

For SDS-PAGE, 50 µg of whole cell lysates in RIPA buffer (Sigma-Aldrich, St. Louis, MO, USA) were denatured in SDS-loading dye (Bio-Rad Laboratories, Hercules, CA, USA) and loaded onto 4–15% denaturing gradient gels (Bio-Rad Laboratories, Hercules, CA, USA). Proteins were transferred onto nitrocellulose membranes, and blots were blocked with 5% non-fat dry milk in PBST, followed by protein-specific antibody incubation overnight at 4 °C. The blots were washed thrice with PBST and incubated with HRP-coupled secondary antibodies. Following three washes with PBST, the blots were developed with SuperSignal West Dura (ThermoFisher, Waltham, MA, USA). For immunoprecipitation, the protein A + G agarose beads were boiled in SDS loading dye and loaded onto a gel, and Western blotting was performed.

### 2.15. Human Cytokine/Chemokine Array

THP-1 culture supernatants or MiaPaCa-2 EMD-CM or THP-1 culture supernatants after 24 h treatment with MiaPaCa-2 EMD-CM were obtained using the Proteome Profiler Human Cytokine Array kit (R&D Systems, Minneapolis, MN, USA), as instructed by the manufacturer. Briefly, cytokine/chemokine array panel membranes spotted with specific antibodies against cytokines/chemokines were incubated with 2 mL of the above-indicated culture supernatants, followed by incubation with detection antibodies, and the membranes were developed with chemiluminescence to detect cytokines/chemokines.

### 2.16. Quantification of Hsp70 in Cancer Cell EMD-CM by ELISA

Cancer cell lines were grown in their respective media until 70% confluency. The cells were washed twice with serum-free media and replenished with serum-free media. After 24 h, EMD-CM was collected. Hsp70 in the EMD-CM was quantified using an Hsp70 ELISA Kit (ThermoFisher, Waltham, MA, USA) according to the manufacturer’s protocol.

### 2.17. Statistical Analysis

All statistical analyses were conducted using GraphPad Prism 6 software (GraphPad Software Inc., San Diego, CA, USA). The data were analyzed using a student-paired *t*-test or ANOVA followed by Bonferroni’s post hoc test. In vitro experiments were performed two to three times. In vivo experiments were conducted at least three times. * *p* < 0.05, ** *p* < 0.01, *** *p* < 0.001, and **** *p* < 0.0001.

## 3. Results

### 3.1. Cancer Cell-Conditioned Media Induce MΦ Differentiation and M2 Polarization

**Figure 1:**
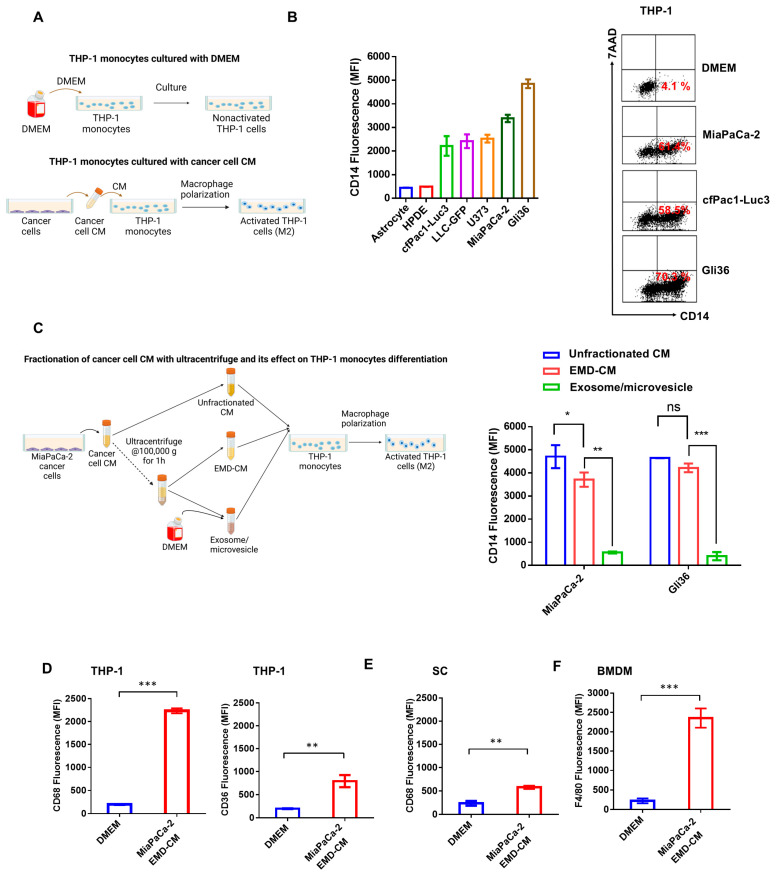
Cancer cells secrete macrophage (MΦ) differentiation factor(s) and are predominantly present in the microparticle/exosome-free fraction of the conditioned media. (**A**) Schematic showing the culture of THP-1 monocytes with DMEM and conditioned media of cancer cells for 24 h. (**B**) Bar graph (**left** panel) and the dot plot (**right** panel) showing the flow cytometric measurement of CD14 expression on THP-1 cells cultured with conditioned media from indicated cancer cell lines. (**C**) Schematic showing the fractionation of cancer cell-conditioned media using ultracentrifugation and THP-1 cells cultured with unfractionated conditioned media, the exosome/microvesicle-depleted (EMD-CM) fraction, and the exosome/microvesicle fraction (**left** panel). Flow cytometry analysis of CD14 expression on THP-1 cells cultured with fractionated supernatant and pellet of conditioned media obtained after ultracentrifugation from MiaPaCa-2 and Gli36 cells (**right** panel). (**D**) Flow cytometric measurement of CD68 (**left** panel) and CD36 (**right** panel) expression on THP-1 cells cultured with MiaPaCa-2 EMD-CM. Flow cytometric analysis of CD68 (**E**) expression on SC MΦ cells and F4/80 (**F**) expression on BMDM cultured with MiaPaCa-2 EMD-CM. The experiments were repeated at least twice. ns: not significant, * *p* < 0.05, ** *p* < 0.01, and *** *p* < 0.001.

Cancer cells escape host immune surveillance via multiple mechanisms, including the generation of immunosuppressive M2 MΦs through tumor-secreted factors [13,14,41]. Therefore, MΦ differentiation of human THP-1 monocyte cells cultured with conditioned media obtained from cancer cell lines was analyzed as illustrated in Figure 1A. Conditioned media from indicated cancer cell lines, including lung, pancreatic, glioblastoma cell lines, and PMA induced robust THP-1 differentiation, as evidenced by CD14 expression (Figure 1B left and right panels, and Figure S1A). In contrast, there was no change in CD14 expression in THP-1 cells cultured with conditioned media from primary human astrocytes or human pancreatic duct epithelial cells (HPDE) (Figure 1B left and right panels).

Before investigating THP-1 activity further, the conditioned media were ultracentrifuged to determine whether any differentiating activity was associated with exosomes/microparticles. The supernatant of ultracentrifuged conditioned media from MiaPaCa-2 and Gli36 cells retained activity. The pellet fraction was mixed with DMEM in an equal volume to the supernatant, and, as shown in Figure 1C, showed no or little activity. Therefore, all subsequent conditioned media studies used EMD-processed samples.

To determine whether the M2 polarization was generalizable to other MΦs populations, we assessed the impact of Gli36 and MiaPaCa-2 EMD-CM on primary MΦs, HMDM and BMDM, tumor-derived MΦs, and J774 cells. The treatment of primary human HMDM with Gli36 EMD-CM resulted in a significant upregulation of M2 markers CD206 and CD163, comparable to the effect induced by established M2 polarizing cytokines IL-4 and IL-13, and substantially greater than the response elicited by the M1 polarizing cytokine IFNγ (Figure S1B). Similarly, Gli36 EMD-CM stimulated the expression of the M2 markers CD206 and CD301 in the mouse macrophage J774 cell line (Figure S1C). Additionally, MiaPaCa-2 EMD-CM induced the expression of the recognized M2 macrophage markers CD36 and CD68 in THP-1 cells (Figure 1D, left and right panels). To evaluate the effects on other cell types, CD68 expression was analyzed in the human monocytic cell line, SC cells (Figure 1E), while F4/80, a pan macrophage marker, was evaluated in BMDM (Figure 1F). MiaPaCa-2 EMD-CM also enhanced the expression of CD68 in SC cells (Figure 1E) and F4/80 in mouse bone marrow cells (Figure 1F). Taken together, these findings indicate that soluble factors present in cancer cell-conditioned media promote MΦs differentiation and M2 polarization in various MΦs populations, including primary MΦs HMDM and BMDM, tumor-derived macrophages represented by the J774 cell line, and monocytic cells such as SC and THP-1 cells.

### 3.2. Identification of Cancer-Secreted Hsp70 as the Causal Factor for MΦ Differentiation

**Figure 2:**
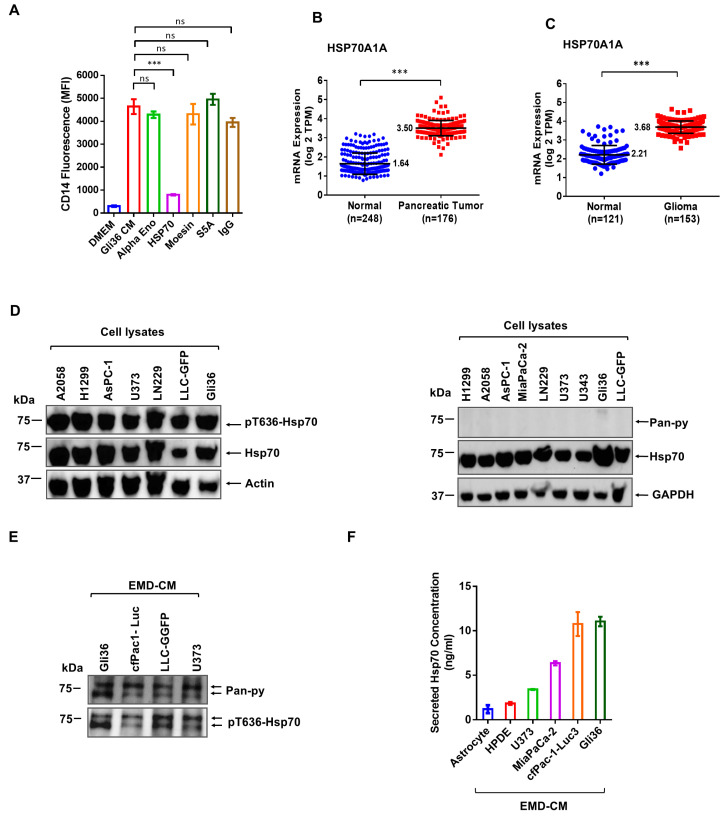
Hsp70 in cancer cell EMD-CM plays a pivotal role in MΦ differentiation. (**A**) Bar graph showing flow cytometric measurement of CD14 expression on THP-1 cells cultured with Gli36 EMD-CM together with indicated mAbs. A total of 200 ng mAbs was used for the neutralization assay. TCGA database showing the HSP70A1A mRNA expression in pancreatic (**B**) and glioma cancer (**C**) cells in comparison to normal cells. (**D**) Western blots of whole-cell lysates from indicated cancer cell lines were probed with anti-pT636-Hsp70 (**top** row), anti-Hsp70 mAb (**middle** row), and anti-actin mAb (**lower** row) (**left** panel). Western blots of whole cell lysates from indicated cancer cell lines were probed with pan-phosphotyrosine (pY) mAb (**top** row), anti-Hsp70 mAb (**middle** row), and/or anti-GAPDH mAb (**bottom** row) (**right** panel). (**E**) Western blots of the EMD-CMs from indicated cancer cell lines were probed with pan-anti-pY mAb (**top** row) and anti-pT636-Hsp70 mAb (**bottom** row). (**F**) Quantification of Hsp70 content in EMD-CM from indicated cancer cell lines using ELISA. The experiments were repeated at least twice. ns: not significant and *** *p* < 0.001. The original WB figure can be found in Figure S4.

To identify the causal factor(s) involved in MΦ differentiation, we performed LC-MS analyses of Gli36 and MiaPaCa-2 EMD-CMs, which showed the highest CD14 activity in THP-1 differentiation (Figure 1B). Among approximately 20 abundant proteins identified in both EMD-CMs, we focused on those known to have immune-regulatory functions, including Hsp70, *α*-enolase, moesin, and S5A. nAbs targeting these individual proteins were used to identify the functional protein in Gli36 EMD-CM. mAbs against Hsp70 showed potent inhibition of Gli36 EMD-CM-induced THP-1 differentiation (Figure 2A). Corroborating its potential importance, the human TCGA data set shows elevated Hsp70 RNA expression in pancreatic cancers and glioma (Figure 2B,C).

To confirm the protein expression of Hsp70, including phosphorylated and unphosphorylated forms, the intracellular and extracellular Hsp70 protein levels in different cancer cell lines were assessed using a Western blot. Robust levels of intracellular Hsp70 were detected using an antibody that recognizes most forms of Hsp70 (Figure 2D, left panel, and right panel). Hsp70 phosphothreonine 636 (Hsp70-pT636) [30] was detected both in intracellular (Figure 2D, left panel) and secreted fractions (Figure 2E). In contrast, Hsp70 pan-phosphotyrosine (Hsp70-Pan-pY) was only present in the EMD-CM as secreted Hsp70 (Figure 2D, right panel, and Figure 2E). Secreted Hsp70s from the conditioned media of human cancer cell lines were also confirmed using a human IgG Hsp70 ELISA kit (Figure 2F). Hsp70 from a commercial source did not demonstrate any effect on monocyte differentiation. Those results were consistent with those of other studies [42,43], as commercial Hsp70 is an intracellular isoform lacking potential post-translational modifications that may be critical for the assessed bioactivity.

These findings demonstrate that Hsp70 is expressed in all the cancer cell lines tested. More specifically, the secreted Hsp70, in contrast to the intracellular form, is tyrosine-phosphorylated, suggesting a possible role for tyrosine phosphorylation in Hsp70 secretion and extracellular actions on MΦ polarization.

### 3.3. Cancer-Secreted Hsp70 Induces MΦ to Secrete Chemokines/Cytokines Implicated in MΦ Recruitment and Polarization

To assess the associated functional consequences of EMD-CM exposure, we measured the chemokine/cytokine profile from THP-1 cells treated with Gli36 EMD-CM. In addition to the expected M2 polarization, treated THP-1 cells secreted not only MCP-1, CCL5, and ICAM-1 (factors linked to both MΦ recruitment and M2 polarization), but also complement factor C5A, which is involved in M2 polarization (Table S1, Figure S2). While our cytokine array results demonstrate that EMD-CM from cancer cells primarily induces M2 polarization in macrophages, we also observed an increase in cytokines traditionally associated with M1 polarization, such as TNF-α, IL-1β, and IL-6 (Table S1, Figure S2). These results indicate that the cytokine production could be a part of a dynamic, dual, or intermediate polarization state. Even though some M1 cytokines are secreted during the immune response, M2 polarization can dominate in response to a robust M2 cytokine release.

### 3.4. Hsp70 Knockdown (KD) in Cancer Cells Impairs Cancer Cell-Induced MΦ Differentiation, Decreases Tumor Growth in Mice, and Alters Intra-Tumor MΦ Polarization

**Figure 3:**
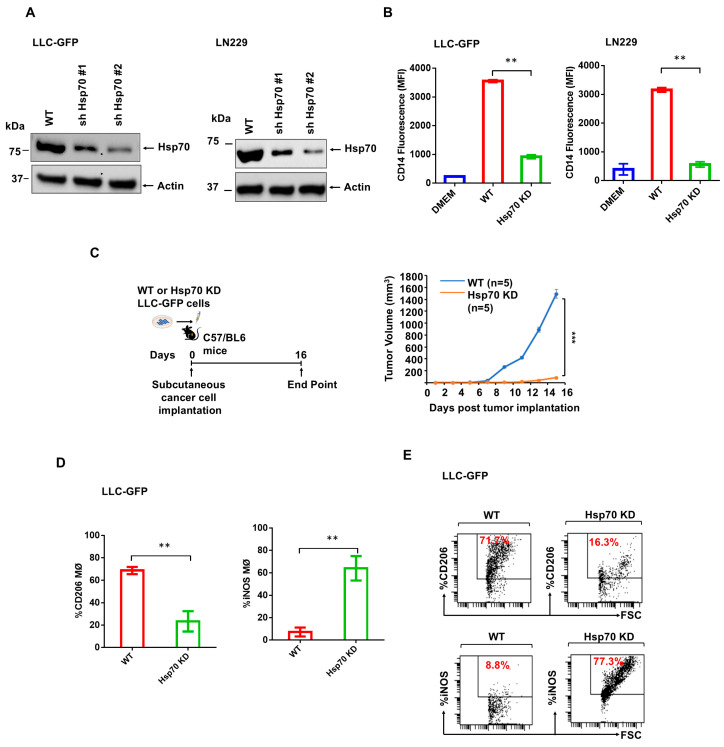
Hsp70 is the causal factor in cancer cell EMD-CM-induced THP-1 differentiation and is required for intra-tumor M2 MΦ maintenance. (**A**) Western blot analyses of whole cell lysates from WT (control shRNA), shHsp70 #1 or shHsp70 #2 from LLC-GFP cells (**left** panel), and LN229 cells (**right** panel) probed with indicated monoclonal antibodies (mAbs). (**B**) CD14 expression on THP-1 cells cultured with conditioned media from WT (control shRNA) or shHsp70 #2 expressing LLC-GFP cells (**left** panel) and LN229 cells (**right** panel), measured using flow cytometry. Schematic showing implantation of subcutaneous tumors from LLC-GFP cells in mouse flank ((**C**), **left** panel). Subcutaneous tumor growth from WT (blue line) or Hsp70 KD LLC-GFP cells (orange line) ((**C**), **right** panel). (**D**) Flow cytometric measurement of CD206 (**left** panel) and iNOS (**right** panel) expression in MΦs isolated from subcutaneous tumors from WT LLC-GFP cells expressing control shRNAs, and Hsp70 KD tumors. (**E**) Flow cytometric dot plots showing the CD206 (**top** panel) and iNOS (**bottom** panel) expression in MΦs isolated from subcutaneous tumors from WT LLC-GFP cells expressing control shRNAs, and Hsp70 KD tumors. The experiments were repeated at least twice. ** *p* < 0.01, and *** *p* < 0.001. The original WB figure can be found in Figure S5.

We further explored the influence of conditioned media-derived Hsp70 on MΦ differentiation and extended the studies to tumor growth and intra-tumor MΦ polarization. We performed Hsp70 knockdown (KD) using the lentiviral-mediated expression of control shRNAs (WT) or Hsp70 targeting shRNAs in two cancer cell lines, LLC-GFP (Figure 3A, left panel) and LN229 (Figure 3A, right panel). In vitro, Hsp70 KD LLC-GFP and LN229 cells were viable and grew normally, comparable to WT cells, most likely because Hsp70 expression was not completely eliminated (Figure 3A, left and right panels). The KD of Hsp70 in LLC-GFP and LN229 cells led to a marked decrease in the MΦ differentiation activity of EMD-CMs derived from Hsp70-KD LLC-GFP compared with EMD-CMs from control shRNA-treated cells (Figure 3B, left and right panels). Notably, when these cells were implanted subcutaneously into C57/BL6 mice (Figure 3C, left panel), tumor growth was significantly impaired from Hsp70 KD LLC-GFP cells compared to tumor growth from LLC-GFP cells treated with control shRNAs (Figure 3C, right panel). Importantly, tumors from LLC-GFP cells with control shRNAs (WT) were enriched with pro-tumorigenic M2-polarized MΦs (CD206) compared to Hsp70 KD tumors (Figure 3D, left and Figure 3E, top left and right panels). On the other hand, WT tumors had lower M1 MΦs (iNOS) than Hsp70 KD tumors (Figure 3D, right and Figure 3E, bottom left and right panels).

### 3.5. Cancer-Secreted Hsp70 Acts Through MΦ TLR2 to Induce MerTK Upregulation

**Figure 4:**
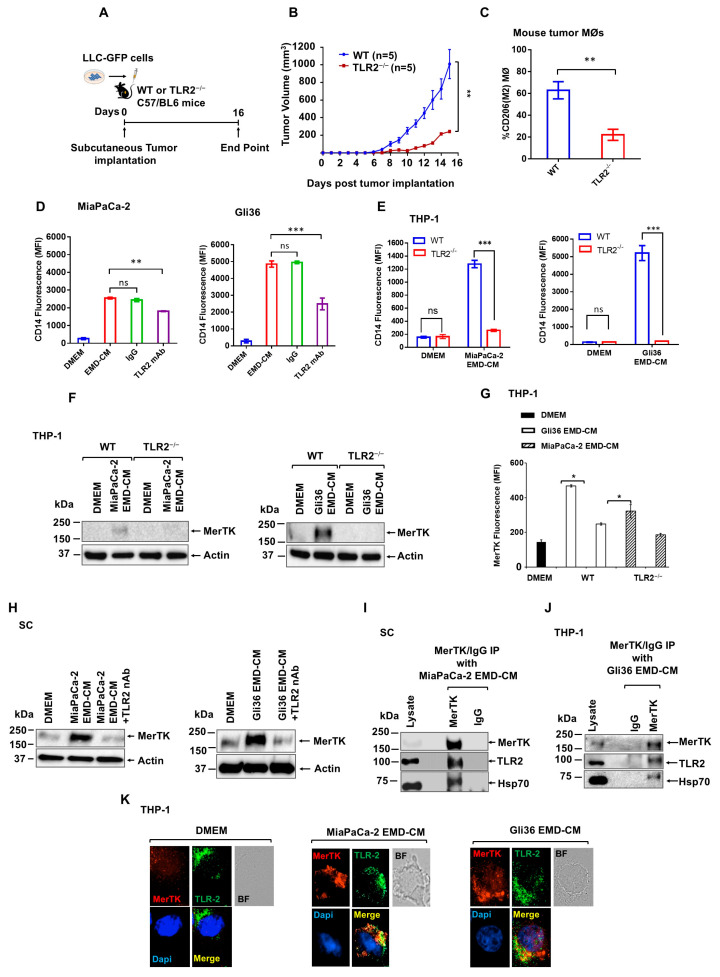
Cancer cell EMD-CMs induce MΦ M2 polarization through TLR2-mediated MerTK expression. (**A**) Schematic showing subcutaneous implantation of LLC-GFP tumor cells into the flanks of WT and TLR2^−/−^ mice and the endpoint of the experiment at 16 days. (**B**) Tumor growth curves of LLC-GFP subcutaneous tumors in WT and TLR2^−/−^ mice. (**C**) Expression of CD206 in tumor MΦs isolated from LLC-GFP subcutaneous tumors in WT and TLR2^−/−^ mice analyzed using flow cytometry. (**D**) Bar graphs showing the flow cytometric analysis of CD14 expression on THP-1 cells cultured in EMD-CMs from MiaPaCa-2 (**left** panel) and Gli36 (**right** panel) in the presence or absence of TLR2 nAbs. DMEM indicates THP-1 cells cultured in DMEM alone. (**E**) Bar graph showing the flow cytometric analyses of CD14 expression in WT and TLR2^−/−^ THP-1 cells cultured in EMD-CMs from MiaPaCa-2 (**left** panel) and Gli36 (**right** panel) cells. Red, green, blue, and yellow colors represent MerTK, TLR-2, nuclei, and co-localized area, respectively. BFs represent bright field images. (**F**) Western blot analyses of the MerTK in WT and TLR2^−/−^ THP-1 cells cultured with DMEM or EMD-CMs from MiaPaCa-2 (**left** panel) and Gli36 (**right** panel) cells. (**G**) Flow cytometric measurement of MerTK expression in WT and TLR2^−/−^ THP-1 cells cultured in EMD-CMs from MiaPaCa-2 and Gli36 cells. (**H**) Western blots of SC MΦ cells cultured with DMEM or EMD-CMs from MiaPaCa-2 (**left** panel) and Gli36 (**right** panel) in the absence/presence of TLR2 nAbs. (**I**) Western blots of MerTK immunoprecipitates from SC cells cultured with MiaPaCa-2 EMD-CM probed with indicated mAbs. (**J**) Western blots of MerTK immunoprecipitates from THP-1 cells cultured with Gli36 EMD-CMs probed with indicated mAbs. (**K**) Immunofluorescent staining of THP-1 cells cultured for 24 h with DMEM (**left** panel), EMD-CMs from MiaPaCa-2 (**middle** panel), and Gli36 (**right** panel). The experiments were repeated at least twice. ns: not significant, * *p* < 0.05, ** *p* < 0.01, and *** *p* < 0.001. The original WB figure can be found in Figure S6.

The MΦ polarization induction of immune regulatory pathways critical for this conversion suggested a direct and robust receptor-based action for Hsp70. Previous studies indicated a functional binding of Hsp70 to TLR2 [32]. First, to test whether TLR2 is involved in tumor growth and MΦ polarization in mice, LLC-GFP were subcutaneously implanted in WT and TLR2^−/−^ mice, and tumor growth and MΦ polarization were monitored (Figure 4A). Tumor growth in TLR2^−/−^ mice was significantly slower compared to tumor growth in WT mice (Figure 4B), and intra-tumor MΦs from TLR2^−/−^ mice showed a significant reduction in M2 polarization compared to MΦs from WT mice (Figure 4C). Next, to assess the impact of TLR2 on monocyte differentiation, we used neutralizing TLR2 nAbs to reduce CD14 expression in THP-1 cells in response to EMD-CMs from MiaPaCa-2 (Figure 4D, left panel), Gli36 (Figure 4D, right panel), and LLC-GFP (Figure S3A). This finding was confirmed using the TLR2^−/−^ THP-1 cells (Figure 4E, left and right panels). To further investigate the TLR2 requirement in vivo for Gli36 EMD-CM-induced MΦ polarization, we injected Gli36 EMD-CM into peritonea of WT or TLR2^−/−^ mice (Figure S3B). After 24 h, we isolated the peritoneal MΦ and analyzed them using flow cytometry (Figure S3B). Gli36 EMD-CM induction of peritoneal MΦ M2 polarization was significantly reduced in TLR2^−/−^ mice compared to WT mice (Figure S3C left and right panels). There was no significant reduction in cells stained using the pan MΦ marker F4/80 (Figure S3D), indicating a specific effect on M2 polarization.

MΦ MerTK has been shown to be required for M2 polarization [38]. MerTK signaling suppresses M1 pro-inflammatory cytokine production and polarizes the MΦ towards the anti-inflammatory M2 phenotype. Since our data indicated that cancer cell EMD-CM induced MΦ M2 polarization, the involvement of MerTK in this process was tested. After incubating THP-1 cells with MiaPaCa-2 and Gli36 EMD-CMs for 24 h, MerTK induction was detected using Western blot (Figure 4F, left and right panels) and flow cytometry (Figure 4G). The functional relationship between TLR2 and MerTK was demonstrated by the absence of MerTK induction in TLR2^−/−^ THP-1 cells (Figure 4F,G). MerTK induction was detected using immunofluorescent imaging of WT THP-1 (Figure S3E, top row), but not in TLR2^−/−^ cells (Figure S3E, bottom row). Also, upregulation of MerTK was observed in SC MΦ cells in response to MiaPaCa-2 or Gli36 EMD-CM, and this was inhibited by TLR2 nAbs (Figure 4H, left and right panels). These findings suggest that TLR2 is required to trigger a MerTK response.

The results indicated that Hsp70 is associated with TLR2 and significantly induces MerTK, prompting studies to assess the functional connection between TLR2 and MerTK. First, the interaction between Hsp70-stimulated TLR2 and the elevated levels of MerTK were assessed. After incubating SC cells with MiaPaCa-2 EMD-CM for 24 h, TLR2 was detected in MerTK immunoprecipitates, indicating an association between TLR2 and MerTK (Figure 4I). In addition to TLR2, Hsp70 was found in the MerTK immunoprecipitates from Gli36 EMD-CM-treated THP-1 cells (Figure 4J). This association was not observed if IgG was used for immunoprecipitation.

Next, the co-localization of TLR2 and MerTK using immunofluorescence imaging of non-permeabilized cells upon induction with MiaPaCa-2 EMD-CM was assessed. Significant membrane co-localization of TLR2 and MerTK in THP-1 cells was noted when treated with MiaPaCa-2 EMD-CM (Figure 4K, middle panel) or Gli36 EMD-CM (Figure 4K, right panel), in contrast to the DMEM control (Figure 4K, left panel). These findings suggest that Hsp70 is secreted by cells and binds to MΦ TLR2, promoting the expression of MerTK, which contributes to MΦ M2 polarization and subsequent immunosuppression.

## 4. Discussion

This study demonstrates that cancer-secreted Hsp70 induces a novel immunosuppressive signaling mechanism between cancer cells and MΦ. Cancer-secreted Hsp70 triggered immunosuppression through TLR2-dependent upregulation of MerTK receptors and may require an Hsp70-TLR2-MerTK interaction. The secreted Hsp70 was tyrosine- and threonine-phosphorylated, whereas tyrosine-phosphorylated Hsp70 was not detectable in cell lysates, suggesting that tyrosine phosphorylation may play a role in Hsp70 secretion and function. Further supporting a role for Hsp70, TLR2, and MerTK, the tumors from Hsp70 KD LLC-GFP and LN229 cells, and LLC-GFP tumors from TLR2 null mice showed impaired MΦ polarization and tumor growth. As a role for secreted Hsp70 in MΦ M2 polarization has not been described previously, the finding that cancer-secreted Hsp70 controls MΦ polarization through TLR2 and MerTK receptors establishes a new immunosuppressive molecular communication mechanism between cancer cells and MΦ mediated by Hsp70.

The identification of cancer-secreted Hsp70 as a potent regulator of immunosuppressive M2 polarization in the TME adds to the known factors influencing M2 polarization. Even though a variety of immunoregulatory proteins were detected in cancer cell EMD-CM using LC-MS analyses, such as enolase, moesin, and S5A, neutralization experiments revealed that Hsp70 appeared to be the only species with a potent MΦ differentiation ability under these assays’ conditions.

This study’s most important finding, besides identifying a new role for Hsp70, is the involvement of the MΦ TLR2 receptor pathway. Seemingly conflicting results have been previously reported, showing Hsp70 immunomodulatory functions that are both pro-inflammatory and anti-inflammatory [44,45]. While the TLRs are primarily known for pro-inflammatory functions, our study identified the engagement of TLR2 receptors by cancer-secreted Hsp70 triggers anti-inflammatory MΦ M2 polarization through the induction of MerTK receptors on MΦs, followed by the association of Hsp70, TLR2, and MerTK. MerTK receptors, belonging to the TAM family of receptors, play essential roles in immune homeostasis, tissue repair, and MΦ M2 polarization [37,38]. Thus, our data establish a novel cancer-induced functional connection between TLR2 and MerTK receptors on MΦ. The definitive characterization of the nature of the complex formation with TLR2 and MerTK, and the subsequent MΦ M2 polarization and the role of phosphorylation will require the purification of an active phosphorylated form of Hsp70 from conditioned media.

Intracellular Hsp70 in cancer is speculated to have increased phosphorylation [28]. The phosphorylation of Hsp70 enables increased binding to co-chaperones, and Hsp70 phosphorylation has been linked to increased cellular proliferation [28,29]. Although cancer-secreted Hsp70 is tyrosine-phosphorylated, we could not detect tyrosine-phosphorylated intracellular Hsp70; this may be due to low levels of intracellular py-Hsp70 or the immediate secretion of py-Hsp70. However, the specific phosphorylation type and site(s) involved in TLR2 interaction and MΦ polarization need to be determined. Site-specific mutagenesis of tyrosine phosphorylation sites may help identify the critical phosphorylation site(s) involved in these processes.

Both Hsp70 and TLR2 were detected through immunoprecipitation with MerTK mAbs and were co-localized with MerTK on the membranes of THP-1/SC cells. However, it is not clear how these molecules interact, the nature of interaction domains, or the precise functional consequences of this complex formation. Also, the downstream signaling pathways linking TLR2 and MerTK need to be further elucidated.

Cancer-associated fibroblasts (CAFs) are known to be one of the essential components of the TME. Activated CAFs can promote tumor growth, angiogenesis, invasion, and metastasis, along with extracellular matrix (ECM) remodeling [46]. It is also known that cancer cells and CAFs could induce monocyte-derived macrophages into M2-polarized macrophages [47], but it is not known whether cancer-secreted Hsp70 plays a role in the activation of CAFs. The effect of cancer-secreted Hsp70 on the activation of CAFs, angiogenic pathways, and cytotoxic T-cells needs to be further elucidated.

## 5. Conclusions

Our data indicate that cancer cells secrete Hsp70, which acts on MΦ through TLR2 to upregulate MerTK and promote M2 polarization, a previously unknown functional connection between the TLR2 and MerTK pathways. This study revealed a potent and unexpected role for secreted Hsp70 in the TME, suggesting the presence of a similar immunosuppressive mechanism in different types of cancers. Immunotherapeutic strategies that block the interaction sites between Hsp70, TLR2, and MerTK could be advantageous for cancer treatment by disrupting the immunosuppressive communication between tumor cells and immune cells. Thus, future studies will focus on developing strategies to prevent the formation of this complex and evaluate the feasibility, specificity, and safety of disrupting this axis in the context of cancer treatment.

## Data Availability

No new data sets were created that are posted.

## Supplementary Materials

The following supporting information can be downloaded at: https://www.mdpi.com/article/10.3390/cancers17030450/s1, Figure S1: Cancer cell EMD-CM induces MΦ M2 polarization; Figure S2: Human cytokine array in response to Gli36 EMD-CM; Figure S3: TLR2 is required for the cancer EMD-CM-induced MΦ M2 polarization; Figure S4: The original WB figure of Figure 2D,E; Figure S5: The original WB figure of Figure 3A; Figure S6: The original WB figure of Figure 4F,H–J; Table S1: Cancer cell EMD-CM-induced secretion profile of chemokines/cytokines from THP-1 cells.
