# Supplementary material for: TLR2-Bound Cancer-Secreted Hsp70 Induces MerTK-Mediated Immunosuppression and Tumorigenesis in Solid Tumors
Source: Cancers (Basel). 2025 Jan 28;17(3):450. doi: 10.3390/cancers17030450 (PMC11815864; doi:10.3390/cancers17030450)
Supplement: Supplementary file 1 [file cancers-17-00450-s001.zip › Figure S1.pptx]

## Slide 1
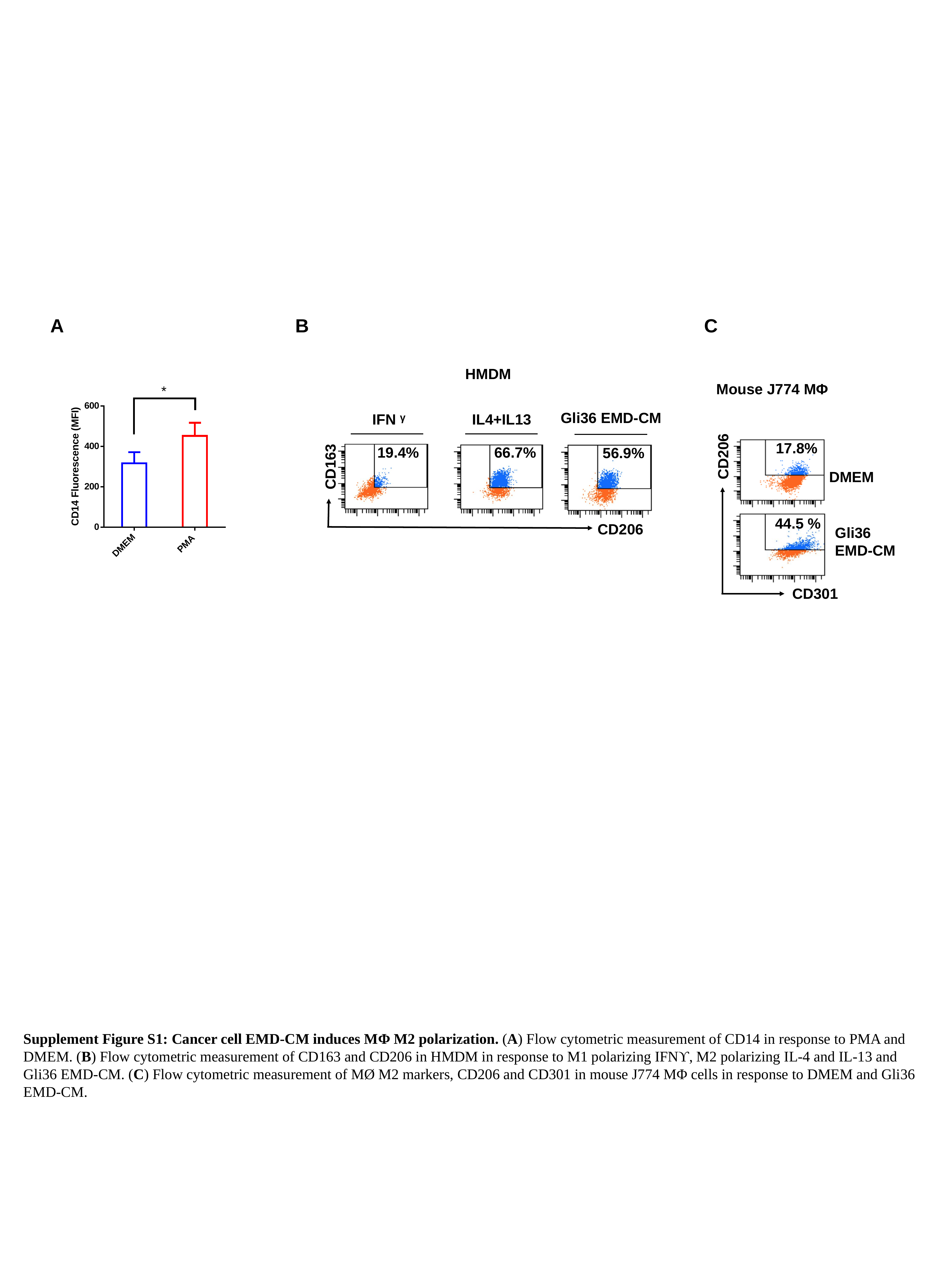

A
B
C
HMDM
Mouse J774 MΦ
Gli36 EMD-CM
IFN ᵞ
IL4+IL13
19.4%
66.7%
56.9%
17.8%
CD206
CD163
DMEM
44.5 %
CD206
Gli36
EMD-CM
CD301
Supplement Figure S1: Cancer cell EMD-CM induces MΦ M2 polarization. (A) Flow cytometric measurement of CD14 in response to PMA and DMEM. (B) Flow cytometric measurement of CD163 and CD206 in HMDM in response to M1 polarizing IFNϒ, M2 polarizing IL-4 and IL-13 and Gli36 EMD-CM. (C) Flow cytometric measurement of MØ M2 markers, CD206 and CD301 in mouse J774 MΦ cells in response to DMEM and Gli36 EMD-CM.
