# Supplementary material for: TLR2-Bound Cancer-Secreted Hsp70 Induces MerTK-Mediated Immunosuppression and Tumorigenesis in Solid Tumors
Source: Cancers (Basel). 2025 Jan 28;17(3):450. doi: 10.3390/cancers17030450 (PMC11815864; doi:10.3390/cancers17030450)
Supplement: Supplementary file 1 [file cancers-17-00450-s001.zip › Figure S2.pptx]

## Slide 1
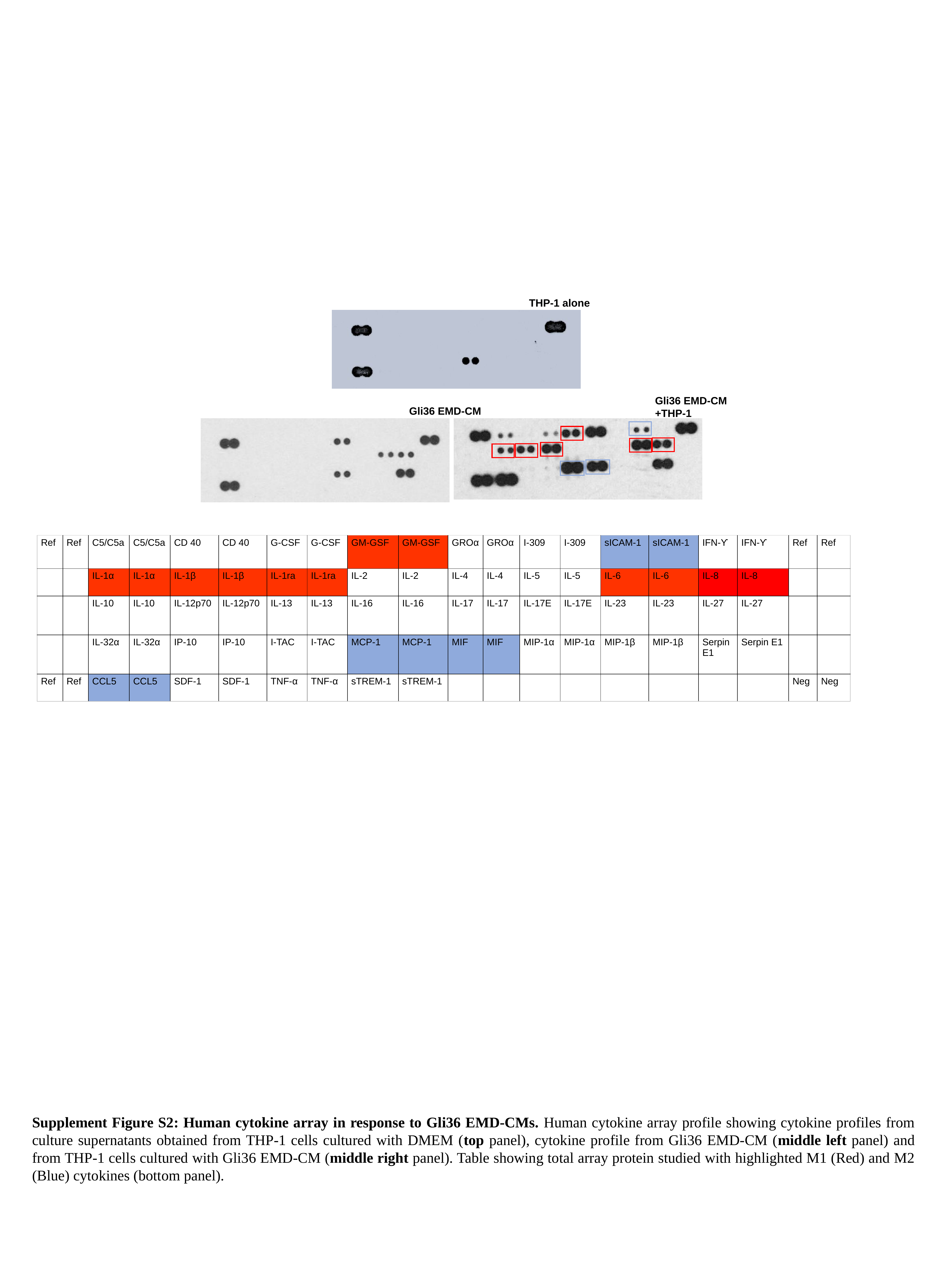

THP-1 alone
Gli36 EMD-CM +THP-1
Gli36 EMD-CM
CXCL1
| Ref | Ref | C5/C5a | C5/C5a | CD 40 | CD 40 | G-CSF | G-CSF | GM-GSF | GM-GSF | GROα | GROα | I-309 | I-309 | sICAM-1 | sICAM-1 | IFN-ϒ | IFN-ϒ | Ref | Ref |
| --- | --- | --- | --- | --- | --- | --- | --- | --- | --- | --- | --- | --- | --- | --- | --- | --- | --- | --- | --- |
| | | IL-1α | IL-1α | IL-1β | IL-1β | IL-1ra | IL-1ra | IL-2 | IL-2 | IL-4 | IL-4 | IL-5 | IL-5 | IL-6 | IL-6 | IL-8 | IL-8 | | |
| | | IL-10 | IL-10 | IL-12p70 | IL-12p70 | IL-13 | IL-13 | IL-16 | IL-16 | IL-17 | IL-17 | IL-17E | IL-17E | IL-23 | IL-23 | IL-27 | IL-27 | | |
| | | IL-32α | IL-32α | IP-10 | IP-10 | I-TAC | I-TAC | MCP-1 | MCP-1 | MIF | MIF | MIP-1α | MIP-1α | MIP-1β | MIP-1β | Serpin E1 | Serpin E1 | | |
| Ref | Ref | CCL5 | CCL5 | SDF-1 | SDF-1 | TNF-α | TNF-α | sTREM-1 | sTREM-1 | | | | | | | | | Neg | Neg |
Supplement Figure S2: Human cytokine array in response to Gli36 EMD-CMs. Human cytokine array profile showing cytokine profiles from culture supernatants obtained from THP-1 cells cultured with DMEM (top panel), cytokine profile from Gli36 EMD-CM (middle left panel) and from THP-1 cells cultured with Gli36 EMD-CM (middle right panel). Table showing total array protein studied with highlighted M1 (Red) and M2 (Blue) cytokines (bottom panel).
