# Supplementary material for: TLR2-Bound Cancer-Secreted Hsp70 Induces MerTK-Mediated Immunosuppression and Tumorigenesis in Solid Tumors
Source: Cancers (Basel). 2025 Jan 28;17(3):450. doi: 10.3390/cancers17030450 (PMC11815864; doi:10.3390/cancers17030450)
Supplement: Supplementary file 1 [file cancers-17-00450-s001.zip › Figure S3.pptx]

## Slide 1
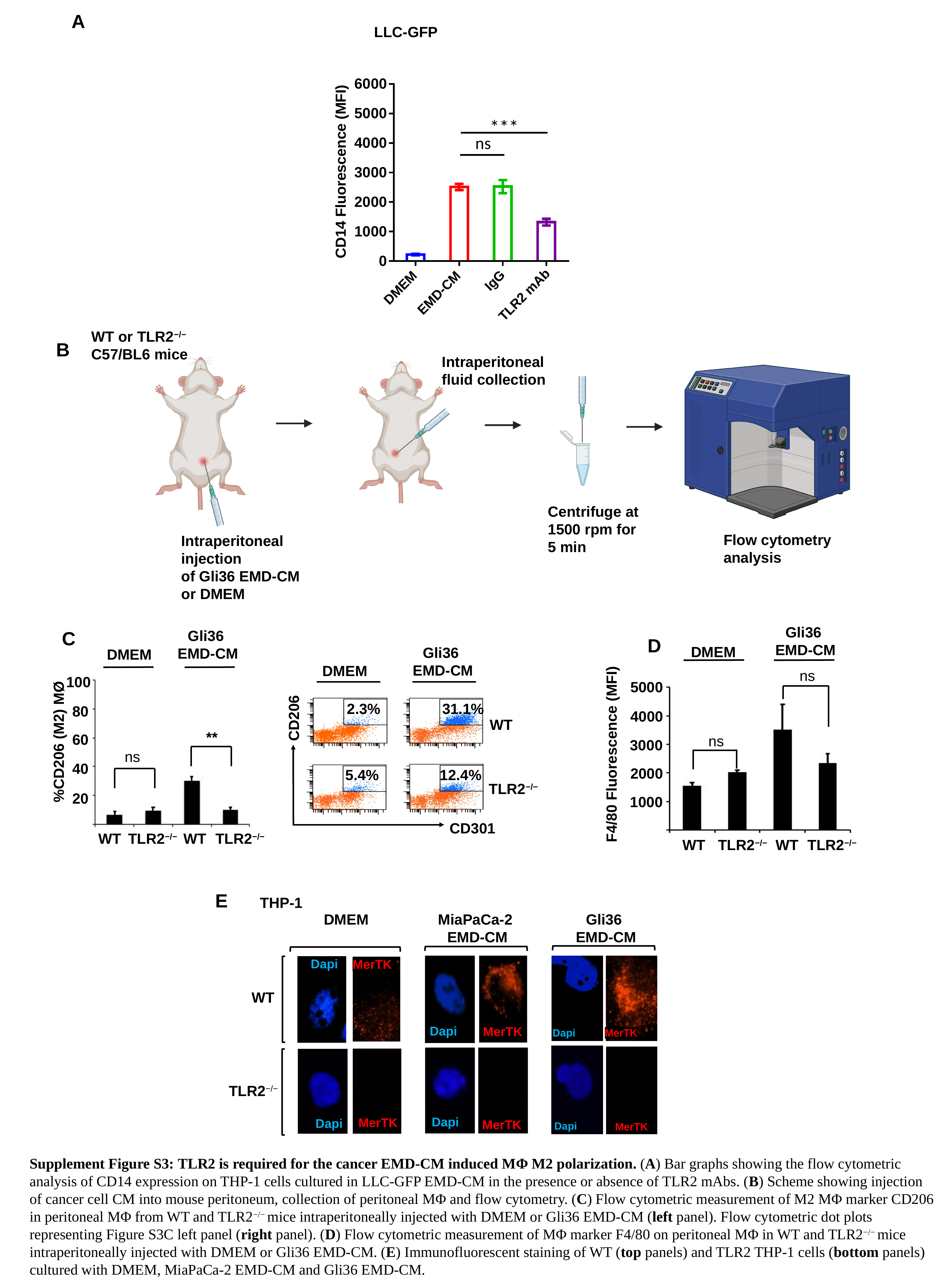

A
LLC-GFP
WT or TLR2−/−
C57/BL6 mice
Intraperitoneal
fluid collection
Centrifuge at
1500 rpm for
5 min
Flow cytometry
analysis
Intraperitoneal
injection
of Gli36 EMD-CM
or DMEM
B
Gli36
EMD-CM
C
Gli36
EMD-CM
DMEM
100
80
**
60
%CD206 (M2) MØ
ns
40
20
WT
TLR2−/−
WT
TLR2−/−
D
DMEM
Gli36
EMD-CM
DMEM
2.3%
31.1%
CD206
WT
5.4%
12.4%
TLR2−/−
CD301
5000
4000
3000
F4/80 Fluorescence (MFI)
2000
1000
ns
ns
WT
WT
TLR2−/−
TLR2−/−
E
THP-1
DMEM
MiaPaCa-2
EMD-CM
Gli36
EMD-CM
Dapi
MerTK
 WT
Dapi
MerTK
Dapi
MerTK
TLR2−/−
Dapi
MerTK
Dapi
MerTK
Dapi
MerTK
Supplement Figure S3: TLR2 is required for the cancer EMD-CM induced MΦ M2 polarization. (A) Bar graphs showing the flow cytometric analysis of CD14 expression on THP-1 cells cultured in LLC-GFP EMD-CM in the presence or absence of TLR2 mAbs. (B) Scheme showing injection of cancer cell CM into mouse peritoneum, collection of peritoneal MΦ and flow cytometry. (C) Flow cytometric measurement of M2 MΦ marker CD206 in peritoneal MΦ from WT and TLR2−/− mice intraperitoneally injected with DMEM or Gli36 EMD-CM (left panel). Flow cytometric dot plots representing Figure S3C left panel (right panel). (D) Flow cytometric measurement of MΦ marker F4/80 on peritoneal MΦ in WT and TLR2−/− mice intraperitoneally injected with DMEM or Gli36 EMD-CM. (E) Immunofluorescent staining of WT (top panels) and TLR2 THP-1 cells (bottom panels) cultured with DMEM, MiaPaCa-2 EMD-CM and Gli36 EMD-CM.
