# Supplementary material for: TLR2-Bound Cancer-Secreted Hsp70 Induces MerTK-Mediated Immunosuppression and Tumorigenesis in Solid Tumors
Source: Cancers (Basel). 2025 Jan 28;17(3):450. doi: 10.3390/cancers17030450 (PMC11815864; doi:10.3390/cancers17030450)
Supplement: Supplementary file 1 [file cancers-17-00450-s001.zip › Figure S4.pptx]

## Slide 1
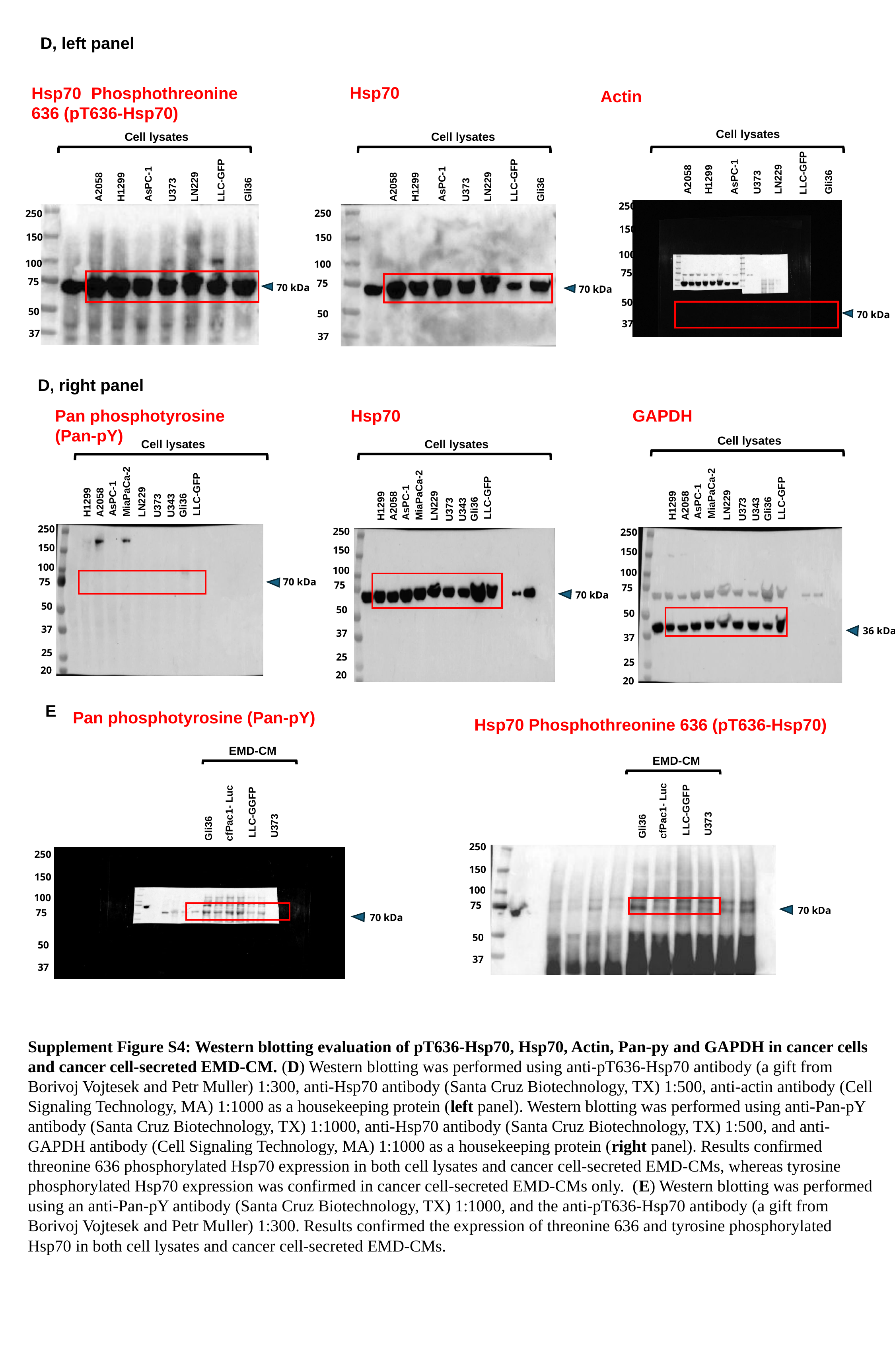

D, left panel
Hsp70
LLC-GFP
AsPC-1
LN229
A2058
H1299
Gli36
U373
250
150
100
75
50
37
70 kDa
Hsp70 Phosphothreonine 636 (pT636-Hsp70)
Actin
LLC-GFP
AsPC-1
LN229
A2058
H1299
Gli36
U373
250
150
100
75
50
37
70 kDa
LLC-GFP
AsPC-1
LN229
A2058
H1299
Gli36
U373
250
150
100
75
50
37
70 kDa
Cell lysates
Cell lysates
Cell lysates
D, right panel
Hsp70
GAPDH
Pan phosphotyrosine
(Pan-pY)
Cell lysates
Cell lysates
Cell lysates
MiaPaCa-2
LLC-GFP
AsPC-1
LN229
H1299
A2058
Gli36
U373
U343
250
150
100
75
50
37
25
20
36 kDa
MiaPaCa-2
LLC-GFP
AsPC-1
LN229
H1299
A2058
Gli36
U373
U343
250
150
100
75
70 kDa
50
37
25
20
MiaPaCa-2
LLC-GFP
AsPC-1
LN229
H1299
A2058
Gli36
U373
U343
250
150
100
75
50
37
25
20
70 kDa
E
Pan phosphotyrosine (Pan-pY)
Hsp70 Phosphothreonine 636 (pT636-Hsp70)
EMD-CM
EMD-CM
cfPac1- Luc
LLC-GGFP
U373
Gli36
250
150
100
75
50
37
70 kDa
LLC-GGFP
U373
Gli36
250
150
100
75
50
37
cfPac1- Luc
70 kDa
Supplement Figure S4: Western blotting evaluation of pT636-Hsp70, Hsp70, Actin, Pan-py and GAPDH in cancer cells and cancer cell-secreted EMD-CM. (D) Western blotting was performed using anti-pT636-Hsp70 antibody (a gift from Borivoj Vojtesek and Petr Muller) 1:300, anti-Hsp70 antibody (Santa Cruz Biotechnology, TX) 1:500, anti-actin antibody (Cell Signaling Technology, MA) 1:1000 as a housekeeping protein (left panel). Western blotting was performed using anti-Pan-pY antibody (Santa Cruz Biotechnology, TX) 1:1000, anti-Hsp70 antibody (Santa Cruz Biotechnology, TX) 1:500, and anti-GAPDH antibody (Cell Signaling Technology, MA) 1:1000 as a housekeeping protein (right panel). Results confirmed threonine 636 phosphorylated Hsp70 expression in both cell lysates and cancer cell-secreted EMD-CMs, whereas tyrosine phosphorylated Hsp70 expression was confirmed in cancer cell-secreted EMD-CMs only. (E) Western blotting was performed using an anti-Pan-pY antibody (Santa Cruz Biotechnology, TX) 1:1000, and the anti-pT636-Hsp70 antibody (a gift from Borivoj Vojtesek and Petr Muller) 1:300. Results confirmed the expression of threonine 636 and tyrosine phosphorylated Hsp70 in both cell lysates and cancer cell-secreted EMD-CMs.
