# Supplementary material for: TLR2-Bound Cancer-Secreted Hsp70 Induces MerTK-Mediated Immunosuppression and Tumorigenesis in Solid Tumors
Source: Cancers (Basel). 2025 Jan 28;17(3):450. doi: 10.3390/cancers17030450 (PMC11815864; doi:10.3390/cancers17030450)
Supplement: Supplementary file 1 [file cancers-17-00450-s001.zip › Figure S5.pptx]

## Slide 1
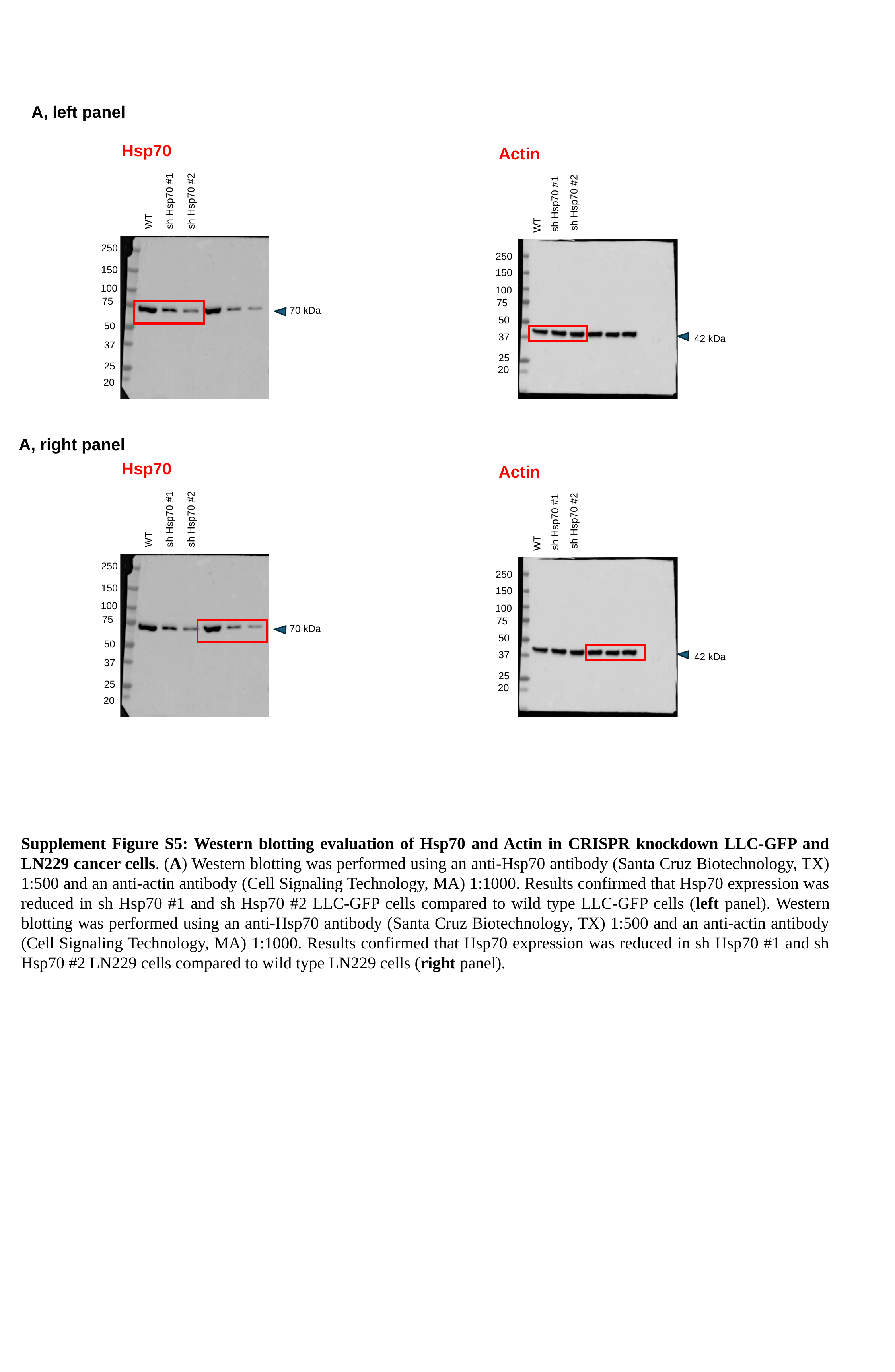

A, left panel
Hsp70
Actin
sh Hsp70 #1
sh Hsp70 #2
WT
250
150
100
75
50
37
25
20
70 kDa
sh Hsp70 #2
sh Hsp70 #1
WT
250
150
100
75
50
37
25
20
42 kDa
A, right panel
Hsp70
Actin
sh Hsp70 #1
sh Hsp70 #2
WT
250
150
100
75
50
37
25
20
70 kDa
sh Hsp70 #2
sh Hsp70 #1
WT
250
150
100
75
50
37
25
20
42 kDa
Supplement Figure S5: Western blotting evaluation of Hsp70 and Actin in CRISPR knockdown LLC-GFP and LN229 cancer cells. (A) Western blotting was performed using an anti-Hsp70 antibody (Santa Cruz Biotechnology, TX) 1:500 and an anti-actin antibody (Cell Signaling Technology, MA) 1:1000. Results confirmed that Hsp70 expression was reduced in sh Hsp70 #1 and sh Hsp70 #2 LLC-GFP cells compared to wild type LLC-GFP cells (left panel). Western blotting was performed using an anti-Hsp70 antibody (Santa Cruz Biotechnology, TX) 1:500 and an anti-actin antibody (Cell Signaling Technology, MA) 1:1000. Results confirmed that Hsp70 expression was reduced in sh Hsp70 #1 and sh Hsp70 #2 LN229 cells compared to wild type LN229 cells (right panel).
