# Supplementary material for: TLR2-Bound Cancer-Secreted Hsp70 Induces MerTK-Mediated Immunosuppression and Tumorigenesis in Solid Tumors
Source: Cancers (Basel). 2025 Jan 28;17(3):450. doi: 10.3390/cancers17030450 (PMC11815864; doi:10.3390/cancers17030450)
Supplement: Supplementary file 1 [file cancers-17-00450-s001.zip › Table S1.pptx]

## Slide 1
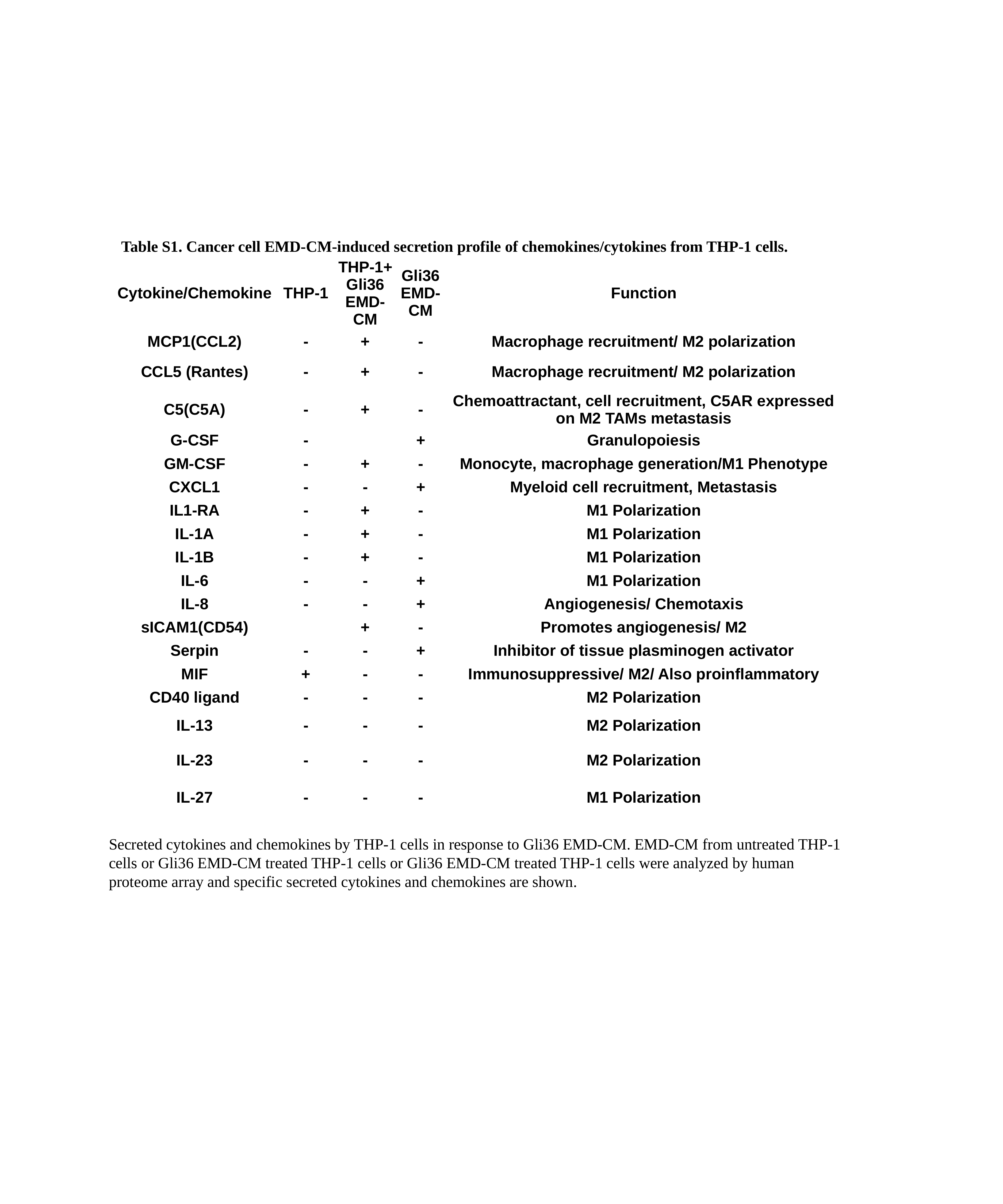

Table S1. Cancer cell EMD-CM-induced secretion profile of chemokines/cytokines from THP-1 cells.
| Cytokine/Chemokine | THP-1 | THP-1+ Gli36 EMD-CM | Gli36 EMD-CM | Function |
| --- | --- | --- | --- | --- |
| MCP1(CCL2) | - | + | - | Macrophage recruitment/ M2 polarization |
| CCL5 (Rantes) | - | + | - | Macrophage recruitment/ M2 polarization |
| C5(C5A) | - | + | - | Chemoattractant, cell recruitment, C5AR expressed on M2 TAMs metastasis |
| G-CSF | - | | + | Granulopoiesis |
| GM-CSF | - | + | - | Monocyte, macrophage generation/M1 Phenotype |
| CXCL1 | - | - | + | Myeloid cell recruitment, Metastasis |
| IL1-RA | - | + | - | M1 Polarization |
| IL-1A | - | + | - | M1 Polarization |
| IL-1B | - | + | - | M1 Polarization |
| IL-6 | - | - | + | M1 Polarization |
| IL-8 | - | - | + | Angiogenesis/ Chemotaxis |
| sICAM1(CD54) | | + | - | Promotes angiogenesis/ M2 |
| Serpin | - | - | + | Inhibitor of tissue plasminogen activator |
| MIF | + | - | - | Immunosuppressive/ M2/ Also proinflammatory |
| CD40 ligand | - | - | - | M2 Polarization |
| IL-13 | - | - | - | M2 Polarization |
| IL-23 | - | - | - | M2 Polarization |
| IL-27 | - | - | - | M1 Polarization |
Secreted cytokines and chemokines by THP-1 cells in response to Gli36 EMD-CM. EMD-CM from untreated THP-1 cells or Gli36 EMD-CM treated THP-1 cells or Gli36 EMD-CM treated THP-1 cells were analyzed by human proteome array and specific secreted cytokines and chemokines are shown.
